# Doubling of Decipher Biopsy Genomic Score Is Related to Disease Reclassification on Subsequent Surveillance Biopsy but Not Adverse Features on Radical Prostatectomy

**DOI:** 10.1155/2021/2687416

**Published:** 2021-03-17

**Authors:** Kamyar Ghabili, Nathan Paulson, Jamil S. Syed, Cayce B. Nawaf, Ghazal Khajir, Darryl T. Martin, John Onofrey, Michael S. Leapman, Angelique Levi, Jeffrey C. Weinreb, Peter A. Humphrey, Preston C. Sprenkle

**Affiliations:** ^1^Department of Urology, Yale University School of Medicine, New Haven, CT, USA; ^2^Department of Pathology, Yale University School of Medicine, New Haven, CT, USA; ^3^Department of Radiology and Biomedical Imaging, Yale University School of Medicine, New Haven, CT, USA

## Abstract

The utility of serial Decipher biopsy scores in a true active surveillance population is still unknown. In a man on active surveillance for low-risk prostate cancer, a doubling of the Decipher biopsy score within genomic low-risk category from first to the second biopsy related to biopsy reclassification to Gleason grade group 4 on the third biopsy. However, the final pathology at radical prostatectomy showed Gleason grade group 2 with an organ-confined disease. This case suggests that the genomic risk category of Decipher biopsy scores during active surveillance may be more informative than either the interval genomic score change or the biopsy Gleason grade group.

## 1. Introduction

Active surveillance (AS) is a management option to defer immediate treatment for eligible patients with low-risk prostate cancer while monitoring disease progression [1]. However, distinguishing indolent versus aggressive tumor in patients on AS is challenging [2, 3]. Therefore, apart from repeat biopsies and prostate-specific antigen (PSA) measurements during surveillance, various serum, imaging, and genomic biomarkers are being used for better risk stratification of those patients [4].

The Decipher biopsy test is a genomic classifier that has been independently associated with metastasis, prostate-cancer-specific mortality, and adverse pathology at radical prostatectomy (RP) [2]. Nonetheless, the prognostic merit of the Decipher biopsy test in the true AS population and how the Decipher score changes over time on serial surveillance biopsies is still immature [4]. Here, we present a patient on AS for low-risk prostate cancer whose Decipher biopsy score doubling was related to disease reclassification on subsequent biopsy but not adverse features on radical prostatectomy.

## 2. Case Presentation

The patient is a Scandinavian male presented in 2016 at age 73 due to an elevated serum PSA of 6.3 ng/mL. His urologic history was notable for benign prostatic hyperplasia since 2014 managed with tamsulosin. He denied any family history of prostate cancer or any prior prostate biopsy. On digital rectal examination, no nodule/induration was palpable (clinical T1c). Following a negative multiparametric magnetic resonance imaging (mpMRI) with a prostate volume of 79 mL (PSA density = 0.08), he underwent 12-core transrectal ultrasound- (TRUS-) guided biopsy which showed Gleason grade group (GG) 1 prostate adenocarcinoma involving 80% and 5% of two cores. The Decipher prostate biopsy score (GenomeDx Biosciences, San Diego, CA) was 0.15 which was within genomic low risk and conveyed a 10% risk of high-grade disease at RP. After counseling with his provider for the diagnosed NCCN low-risk prostate cancer, the patient elected to undergo monitoring on AS.

**Figure 1:**
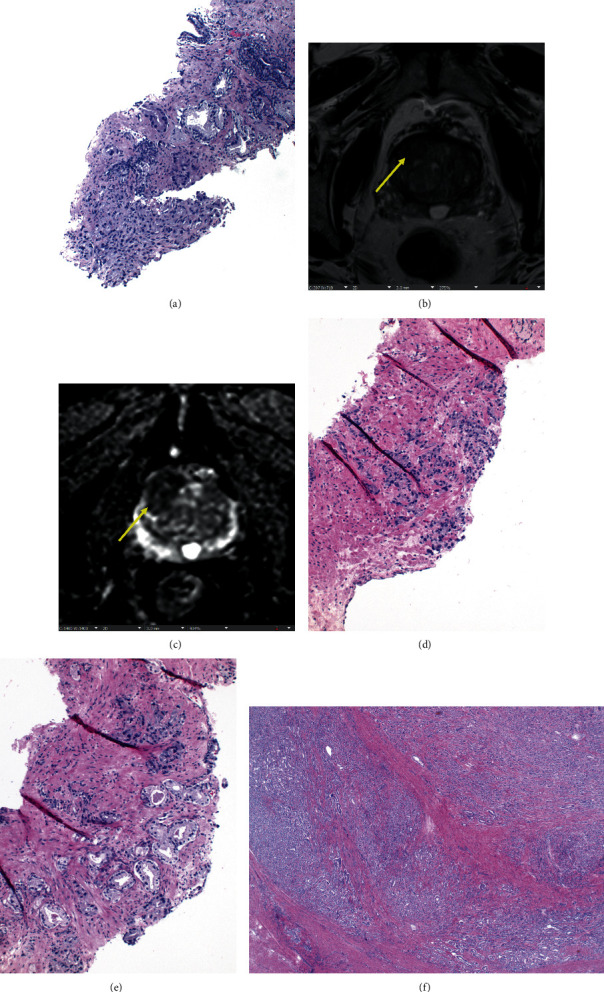
(a) A focus of Gleason GG2 prostatic adenocarcinoma on a targeted core in the 2^nd^ biopsy; (b) T2-weighted image shows lenticular homogeneous moderately hypointense lesion (arrow) measuring 1.5 cm located in right anterior transitional zone in midgland, representative of Prostate Imaging Reporting and Data System category 5; (c) apparent diffusion coefficient map shows marked focal hypointensity (arrow); (d) a single focus of Gleason GG4 prostatic adenocarcinoma on a systematic core in the 3^rd^ biopsy; (e) infiltrative growth of Gleason GG3 prostatic adenocarcinoma on a systematic core in the 3^rd^ biopsy. Gleason pattern 3 is comprised of well-formed glands with relatively clear cytoplasm, small nuclei, and inconspicuous nucleoli. Gleason pattern 4 is made up of smaller, poorly formed glands with amphophilic cytoplasm, larger nuclei, and variably prominent nucleoli; (f) a radical prostatectomy specimen showing two distinct patterns of prostatic adenocarcinoma (the majority of Gleason pattern 3 and small population of pattern 4).

His surveillance mpMRI one year from diagnosis illustrated a 91 mL prostate (PSA density = 0.06) with a 1.5 cm lesion in the right midgland to apex transition zone with Prostate Imaging Reporting and Data System (PI-RADS) score 5 but without extracapsular extension. A right-sided bulge was also palpable on his rectal exam (cT2a). Subsequent mpMRI/TRUS fusion biopsy revealed adenocarcinoma with Gleason GG2 (10% pattern 4) in 75% of a targeted core (Figure 1(a)) and Gleason GG1 in three other targeted cores with the highest involvement of 55%. The Decipher test performed on the biopsy specimen of the core with Gleason GG2 resulted in a score of 0.36 (genomic low risk but with an increased risk of high-grade disease at RP to 17.6%). At this time, the patient elected to stay on AS.

Follow-up serum biomarker, biopsy genomic, and prostate imaging data are listed in Table 1. A repeat mpMRI in 2018 demonstrated a stable 1.5 cm PI-RADS 5 lesion within the transition zone (Figures 1(b) and 1(c)). A simultaneous serum testing revealed a PSA of 9.1 ng/mL with 13% free PSA and prostate health index (PHI) of 47.3. The patient underwent a surveillance mpMRI/TRUS fusion biopsy which showed extensive cancer in 8 of total 17 cores. Five of 12 systematic cores were positive with the maximum Gleason GG4 (one core, Figure 1(d)), followed by Gleason GG3 (one core, Figure 1(e)), and Gleason GG2 (two cores). Three targeted biopsy cores showed Gleason GG1 disease. Preoperative whole body ^99m^Tc-methylene diphosphonate bone scan demonstrated no uptake suspicious for osseous metastasis. The patient underwent robotic-assisted laparoscopic radical prostatectomy and bilateral pelvic lymph node dissection with pathology demonstrating Gleason GG2 acinar adenocarcinoma involving 20% of the gland (Figure 1(f)), positive perineural invasion, negative surgical margins, lymphovascular invasion, extracapsular extension, and 36 negative nodes (pT2 pN0).

## 3. Discussion

This case illustrated that a doubling of the Decipher score (yet within the genomic low-risk category) or Gleason GG4 on preoperative biopsy was not associated with any adverse features such as primary Gleason pattern 4 and/or pT3 in the final pathology. This suggests that the genomic risk category of Decipher biopsy scores during AS may be more informative than either interval genomic score change or biopsy Gleason GG. To the best of our knowledge, this case represents the first in which the relationship between changes of Decipher biopsy score over time and Gleason GG upgrade on biopsy and RP is reported. Nevertheless, studies of a larger cohort of patients on AS with serial Decipher biopsy scores are required before any conclusions can be made in terms of clinical decision-making.

In the present case, we observed a significant elevation in Decipher biopsy score on serial biopsies. By contrast, Cedars and colleagues found a relatively stable Oncotype DX Genomic Prostate Score (GPS) over time during AS. Given that GPS at baseline, but not follow-up, was associated with an upgrade at second biopsy, they concluded that serial genomic testing might be of limited utility in the AS [5]. The present case highlighted the need for a similar study with the Decipher biopsy score as our observation was in favor of a potential association between elevation of the Decipher score and biopsy grade reclassification in the AS setting.

Several other biomarkers are also used during AS to predict biopsy progression which triggers for intervention. Although postdiagnostic PSA-based tests (PSA density, % free PSA, and PHI) have been shown to predict later progression, the prognostic utility of PSA kinetics as well as mpMRI in the AS population is debatable [6]. The present case challenges the benefits of PSA density in the surveillance setting, given that his highest PSA density value was 0.10. We were unable to interpret the values of % free PSA and PHI due to unavailable baseline data.

## 4. Conclusion

This case report demonstrates how serial genomic assessments during AS can provide valuable data regarding the final pathology. It is anticipated that combining biopsy genomic risk category assessment with other biomarkers such as PHI during AS would improve patient risk stratification and offer further evidence for rational management approaches in the setting of biopsy grade reclassification.

## Figures and Tables

**Table 1:** Biomarker measures during active surveillance.

	First biopsy (7/2016)	Second biopsy (8/2017)	Third biopsy (12/2018)
PSA (ng/mL)	6.3	5.7	9.1
% free PSA	—	—	13.2
PHI	—	—	47.3
Biopsy	x	x	x
Decipher score	0.15	0.36	—
Prostate volume (mL)	79	91	90
PSA density (ng/mL/mL)	0.08	0.06	0.10
